# First report of pathogenic *Leptospira* spp. isolated from urine and kidneys of naturally infected cats

**DOI:** 10.1371/journal.pone.0230048

**Published:** 2020-03-10

**Authors:** Abdul Rahman Alashraf, Seng Fong Lau, Siti Khairani-Bejo, Kuan Hua Khor, Mokrish Ajat, Rozanaliza Radzi, Muhammad Azri Roslan, Muhammad Sabri Abdul Rahman

**Affiliations:** 1 Department of Veterinary Clinical Studies, Faculty of Veterinary Medicine, Universiti Putra Malaysia, Serdang, Selangor, Malaysia; 2 Department of Veterinary Pathology and Microbiology, Faculty of Veterinary Medicine, Universiti Putra Malaysia, Serdang, Selangor, Malaysia; 3 Department of Veterinary Preclinical Sciences, Faculty of Veterinary Medicine, Universiti Putra Malaysia, Serdang, Selangor, Malaysia; Instituto Butantan, BRAZIL

## Abstract

Leptospirosis is one of the most widespread zoonotic diseases and can infect both humans and animals worldwide. Healthy cat, as a potential source of exposure to humans, are likely underestimated owing to the lack of overt clinical signs associated with *Leptospira* spp. infection in this species. The aim of the study was to determine the exposure, shedding, and carrier status of leptospires in shelter cats in Malaysia by using serological, molecular, and bacteriological methods. For this study, 82 healthy cats from two shelters were sampled. The blood, urine, and kidneys were tested using the microscopic agglutination test (MAT), polymerase chain reaction (PCR), and bacterial culture. On the basis of serological, molecular, and/or culture techniques, the total detection of leptospiral infection was 29.3% (n = 24/82). Through culture techniques, 16.7% (n = 4/24) of the cats that tested positive were carriers with positive kidney cultures, and one cat was culture positive for both urine and kidney. The *Leptospira* spp. isolates were identified as pathogenic *L. interrogans* serovar Bataviae through serological and molecular methods. Through serological techniques, 87.5% (n = 21/24) had positive antibody titers (100–1600) and most of the Bataviae serogroup (n = 19/21). Using PCR, 16.7% (n = 4/24) of cats were shown to have pathogenic *Leptospira* spp. DNA in their urine. Furthermore, three out of four culture positive cats were serology negative. The present study reports the first retrieval of pathogenic leptospires from urine and kidneys obtained from naturally infected cats. The results provide evidence of the potential role of naturally infected cats in the transmission of leptospires. Additionally, leptospiral infection occurs sub-clinically in cats. The culture isolation provides evidence that healthy cats could be reservoirs of leptospiral infection, and this information may promote the development of disease prevention strategies for the cat population.

## Introduction

Leptospirosis is one of the most common infectious diseases that infect humans [1]. Approximately half a million severe cases are reported annually in humans, but a higher actual number is suspected due to the mis- or under-diagnosed cases in many countries [2,3]. Leptospirosis is caused by the pathogenic serovars of the *Leptospira* spp. and currently, more than 250 pathogenic serovars have been identified worldwide [4]. Rodents are the main reservoirs for pathogenic leptospires and can shed the bacteria asymptomatically in their urine throughout their lifetime [5]. Almost every mammal can be exposed to pathogenic *Leptospira* spp. and may become a carrier for life [6]. Subsequently, infected animals may become potential sources of infection over a long period of time when the *Leptospira* spp. persistently colonize the tubular epithelial cells [7]. The bacteria contaminate the environment and water sources. Thus, this exposes humans and animals to infection through the penetration of leptospires from mucous membranes or skin [8].

Studies reported that the frequency of anti-*Leptospira* spp. antibodies among cats worldwide ranged from 4.8% to 48% [9–12]. *L. interrogans* and *L. borgpetersenii* are the two most common circulating species among humans and animals and have been documented serologically in previous studies on cats [7]. However, leptospirosis in cats is a subject of discussion regarding the role of cats as incidental or maintenance hosts of leptospires. Studies to investigate the ability of cats to transmit *Leptospira* spp. in urine have only been conducted in a few countries. Recently, naturally infected cats with leptospires were shown to be able to shed the pathogenic leptospiral DNA in their urine for up to eight months [13]. Currently, the viability of leptospires in naturally infected cat urine is still unknown [14]. Because of their predatory behavior, cats are likely to be infected directly by rats and less likely through water owing to cats’ natural aversion to it [15]. Clinical signs in naturally infected cats are not well-defined, and in experimentally infected cats, the clinical signs of leptospirosis were not apparent most of the time [9,16]. Recently, a study suggested that the incubation of the pathogen in cats would have to be longer to trigger clinical signs [17]. The most common clinical signs were related to kidney injury such as polyuria and polydipsia with anti-*Leptospira* spp. antibodies detection [18]; however, this was not consistent in all studies [19].

As one of the re-emerging zoonotic diseases, leptospirosis in humans is increasing in Malaysia, from 263 cases reported in 2004 to 1,418 cases in 2009, and more than 7,000 cases reported in 2014 [20,21]. Previous studies on leptospirosis in Malaysia focused mainly on humans and domestic animals. Locally and globally, there is a paucity of information regarding leptospiral infection in cats. In Malaysia, cats are the most common companion animal that lives in close proximity to humans. A lack of awareness of responsible pet ownership such as spaying and neutering has led to an increasing stray-cat population. These stray cats eventually end up in shelters. The leptospiral investigation on shelter cats is crucial as these cats may reflect the health status of the stray-cat population. The aim of the study was to use serological, molecular, and bacteriological methods to investigate the exposure, shedding, and carriage status of leptospires in shelter cats in Malaysia.

## Materials and methods

This study was conducted in strict accordance with the approval of the Institutional Animal Care and Use Committee of Universiti Putra Malaysia (AUP-R050/2017). All cats were obtained randomly from two shelters and were eventually euthanized because of the overpopulation in the shelters. After obtaining consent from the shelter managers, cats were recruited and sampled between June 2017 and February 2018 from the two shelters (shelter 1: n = 48; shelter 2: n = 34).

### Sample collection

A total of 3mL of blood was collected from the jugular vein and was then centrifuged at 4,000 rpm for five minutes. Serum samples were stored at –80°C for further study. After the blood sampling, anesthesia was performed intramuscularly by using 5mg/kg Zoletil (Virbac, France). Then, euthanasia was performed by an intra-cardiac injection of 60mg/kg Pentobarbital (Vetoquinol, France). Urine samples were obtained by cystocentesis and cat kidneys were collected aseptically. Blood, urine, and one randomly chosen kidney were evaluated for *Leptospira* spp. infection using the microscopic agglutination test (MAT), polymerase chain reaction (PCR), and culture techniques.

### Diagnostic tests

#### 1. Microscopic agglutination test (MAT)

Serial dilutions of the samples of sera along with a negative control were prepared in sterile flat-bottomed microtiter plate wells. The sera were tested with a titer starting at 1:50 up to 1:1600, with a cut-off antibody titer of ≥ 100. For each well, 96μL phosphate-buffered saline (PBS) and 4μL serum were added, followed by a two-fold dilution. Antigen cultures of 20 leptospiral serovars were tested against each of the sera. In total, 50μL at a density of 1x10^8^ cells/mL of the antigen was added into each well. The flat-bottomed microtiter plates were incubated for two hours at 30°C. The sera were tested against 20 pathogenic serovars (S1 Data) and one saprophytic serovar Patoc (*L. biflexa*; strain Patoc1) was used for control purposes [22]. Dark-field microscopic examination was performed and the respective titers were recorded when an agglutination of 50% or more was observed.

#### 2. Polymerase chain reaction (PCR)

DNA was extracted using the DNeasy Blood & Tissue kit (Qiagen, Germany). Initially, 20μL proteinase K and 200μL AL buffer were added to the urine and blood samples, and the mixture was incubated at 56°C for 60 minutes for urine and 10 minutes for blood. The DNeasy spin column protocol was performed according to the manufacturer’s instructions. DNA was subjected to PCR using two sets of primers; targeting the 16S rRNA for the leptospiral genus and lipL32 for the pathogenic gene, as shown in S2 Data. The primers and leptospiral PCR protocol were implemented as previously described [23]. DNAase-free water was used as a negative control and Pomona DNA was used as a positive control in all PCR reactions. Gel electrophoresis was performed for the PCR products on a 1.5% agarose gel, stained with SYBRSafe DNA Gel stain (Invitrogen, Carlsbad, CA, EUA) for one hour. The stained amplicons were revealed by using an Alpha imager software with GelDoc (Alphalmager™, USA). For further confirmation, the positive PCR products were sent to First Base Laboratories (Selangor, Malaysia) for further DNA sequencing.

#### 3. Culture and isolation of leptospires

Ellinghausen-McCullough-Johnson-Harris (EMJH) medium was used for the isolation of leptospires. In order to minimize bacterial contamination, 5-Fluorouracil (5-FU; 2g/L) was added. One to two drops of blood and urine were inoculated into a set of two duplicates of the fluids in semi-solid tubes during the sampling process. Additionally, 50 mg homogenized tissue (by using a sterile syringe without a needle) of each kidney was suspended in EMJH-Fluorouracil medium overnight between 28°C and 30°C, allowing leptospires to flow out of the tissue. Subsequently, on the second day, 1 mL of the suspension was pipetted into semi-solid tubes. Sub-culturing was conducted once every two weeks for a period of three months. The media were examined under a dark-field microscope for the presence or potential growth of leptospires approximately every seven days. Positive cultures were subjected to purification. Pure isolates, free of contaminants were used for further serological and molecular identification.

#### 4. Characterization of isolates (serological and molecular identification)

The serological identification by microscopic agglutination test (MAT) was performed as described by the World Health Organization [24]; the purified isolates were serologically tested against a panel of monoclonal antibodies (mAbs). Sixteen hyperimmune sera provided by Forensic and Scientific Services, Department of Health, Leptospirosis Reference Laboratory, Queensland, Australia, were used as a panel in this study (S3 Data). The isolates were tested against the reference set; the corresponding serogroup was identified with a positive reaction when a 50% reduction in the number of free leptospires with or without agglutination observed [25]. These samples were recorded as positive with their respective titers.

DNA extraction was performed on 7–14-day-old cultures of isolates using DNeasy Blood & Tissue kit (Qiagen, Germany) and subjected to PCR by using two sets of primers; targeting the 16S rRNA for the leptospiral genus and *lipL*32 for the pathogenic gene. The positive PCR products (targeting a partial region of the 16S rRNA gene) were sent for DNA sequencing. Subsequently, the sequences were subjected to multiple alignments using the Basic Local Alignment Search Tool (BLAST) algorithm on the GenBank database (http://www.ncbi.nih.gov). Using the online databases of the National Centre of Biotechnology Information (NCBI) website, the 16S ribosomal (rRNA) sequences data of 29 leptospiral strains, representing 16 species of genus *Leptospira* (nine pathogenic, five intermediate and three saprophytic) with the *Leptonema illini* strain Habaki (as an outgroup) were used for a phylogenetic tree construction [26]. The Maximum Likelihood Phylogenetic tree method was used based on model General Time Reversible [27]. MEGA7 software was used for the Molecular Evolutionary Genetics [28]. The CLUSTALW program was used for leptospiral strains alignment. The bootstrap confidence level was set as 1,000 to indicate the level of the degree of confidence in inferring the nodes of the locations of the sequences.

## Results

Eighty-two adult domestic short-haired cats from two shelters (Shelter1: n = 48, Shelter2: n = 34) were recruited for this study (51 males and 31 females). Most of the cats appeared emaciated with evidence of feline upper respiratory disease. There was no flooding around the time of sampling and only adult cats were recruited.

### 1. Microscopic agglutination test (MAT)

On the basis of the cut-off titer of 100, 25.6% (n = 21/82) cats were seropositive with titers ranging from 100 to 1,600 against the pathogenic serogroups Bataviae, Javanica, and Ballum. Bataviae was the predominant serogroup that was detected in 19 cats with titers ranging from 100–800, and two cats with Javanica had titers ranging from 200–1,600. Serogroup Ballum was detected in only one cat at a titer of 100 (Table 1). MAT results involving the use of the local isolates revealed that there was no notable difference in the recorded titers among the sera that tested positive; only one positive serum for Bataviae showed higher titers from 200 to 400. No cross reaction was recorded with the sera that were positive for Ballum and Javanica.

**Table 1:** Summary of the results of the cats with positivity in at least one of the used tests for leptospiral infection in this study.

Cats	MAT serogroup / Titre value	PCR Urine	PCR Blood	Culture urine and kidney
1	**Bataviae/ 200**	***L. interrogans***	N	N
2	Bat & Jav/ 100+200*	***L. interrogans***	N	N
3	**Bataviae/ 200**	***L. interrogans***	N	N
4	**Bataviae/ 200**	***L. interrogans***	N	N
5	**Bataviae/ 100**	N	N	N
6	**Bataviae/ 100**	N	N	N
7	**Javanica/ 1600**	N	N	N
8	**Bataviae/ 400**	N	N	N
9	**Bataviae/ 800**	N	N	N
10	**Bataviae/ 800**	N	N	N
11	**Bataviae/ 200**	N	N	N
12	**Bataviae/ 200**	N	N	N
13	**Bataviae/ 200**	N	N	N
14	**Bataviae/ 400**	N	N	N
15	**Bataviae/ 400**	N	N	N
16	**Bataviae/ 400**	N	N	N
17	**Bataviae/ 400**	N	N	N
18	**Ballum/ 100**	N	N	N
19	**Bataviae/ 100**	N	N	N
20	**Bataviae/ 100**	N	***L. biflexa***	N
21	**Bataviae/ 100**	N	***L. biflexa***	**Urine & kidney (Bataviae)**
22	N	N	N	**Kidney (Bataviae)**
23	N	N	N	**Kidney (Bataviae)**
24	N	N	***L. biflexa***	**Kidney (Bataviae)**
25	N	N	***L. biflexa***	N
26	N	N	***L. biflexa***	N
27	N	N	***L. biflexa***	N
28	N	N	***L. biflexa***	N
**n = 24/82 (29.2%) ^+^**	**n = 21/24 (87.5%)**	**n = 4/24 (16.7%)**	**n = 7/82 (8.5%)**	**n = 4/24 (16.7%)**

### 2. Polymerase chain reaction (PCR)

DNA detection of pathogenic leptospires in urine showed a frequency of 4.9% (n = 4/82). The sequences of leptospiral DNA from positive urine samples were blasted on the NCBI database and revealed high identity to pathogenic representatives (*L. interrogans*; high similarity to Bataviae). Blood detection of leptospiral DNA in blood was 8.5% (n = 7/82). The PCR products of the blood samples showed high identity to saprophytic representatives (*L. biflexa*; high similarity to Patoc1). Two of the seven cats showed seropositivity to Bataviae at a titer of 100. None of the seven cats showed antibodies against serogroup Patoc1 (Table 1).

### 3. *Leptospira* culture and species identification

**Fig 1:**
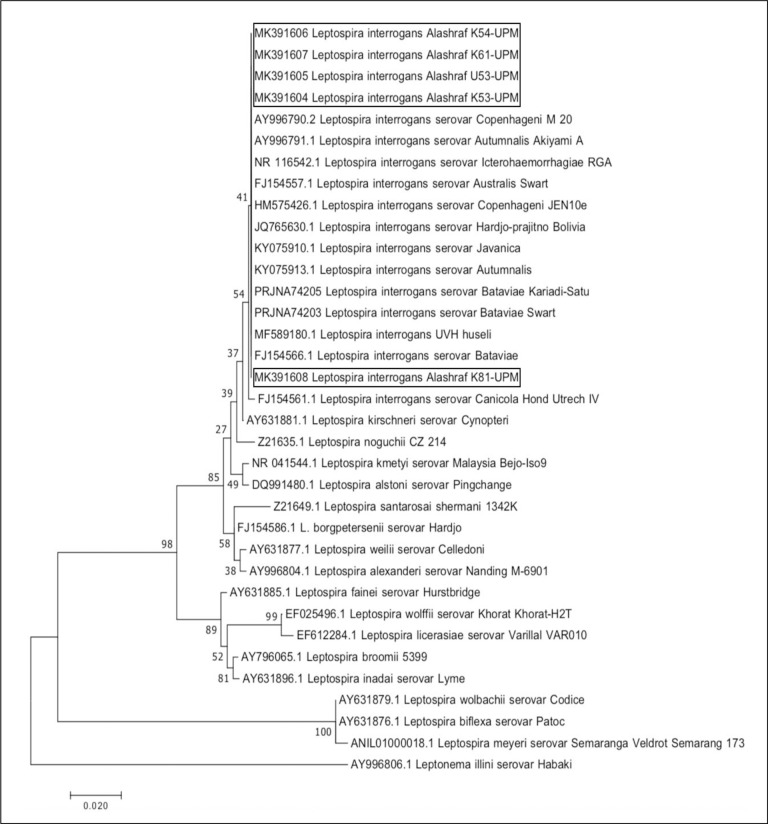
Molecular phylogenetic tree of the leptospiral cats’ isolates using 16S rRNA gene sequencing. The phylogenetic tree presents the gene correlation among the leptospiral cats’ isolates and various *Leptospira* species serovars and strains. *Leptonema* illini serovar Habaki was used for outgrouping. A clear-cut deviation in the sequence of 16S ribosomal (rRNA) gene was denoted in the sequences among the various species of the genus *Leptospira*, the isolates segregated into a distinct clade indicates that all the five isolates from cats are strains of *L. interrogans spp*.

The culture methods revealed leptospiral growth from kidney samples of cats: n = 4/82 (4.9%). Of the four, one cat was culture positive for leptospires in both urine and kidney. All five isolates were identified serologically as serovar Bataviae with titers ranging from 3,200 to 6,400 (Table 2). The isolates were further subjected to PCR and sequencing to confirm the species identification. The isolates were further confirmed through DNA sequencing of the partially amplified 16S rRNA gene sequence to reveal a similarity of 100% with Bataviae strain isolated from other species (*Canis* familiaris) in Malaysia, which was submitted to GenBank previously (100% identity for Bataviae strain, accession number: MF589180.1; *L. interrogans* strain UVH Huseli). The phylogenetic tree (Fig 1) reveals the genetic distance among the cats’ leptospiral isolates and representative strains of *Leptospira* spp. A clear-cut deviation in the sequences of 16S rRNA was denoted among the strains of *Leptospira* spp., and all of the isolates from the five cats were segregated into a distinct clade indicating that all the isolates belonged to *L. interrogans* spp.

**Table 2:** Identification of retrieved isolates from cats’ urine and kidney samples, using microscopic agglutination test (MAT), hyperimmune sera, and polymerase chain reaction (PCR) characterization.

Isolate ID	Identification		Source	Accession number
	MAT; Hyperimmune sera / titre	PCR; 16s rRNA		
Alashraf K53-UPM	Bataviae / 6400	*L. interrogans*	Kidney	MK391604
Alashraf U53-UPM	Bataviae / 3200	*L. interrogans*	Urine	MK391605
Alashraf K54-UPM	Bataviae / 3200	*L. interrogans*	Kidney	MK391606
Alashraf K61-UPM	Bataviae / 3200	*L. interrogans*	Kidney	MK391607
Alashraf K81-UPM	Bataviae / 3200	*L. interrogans*	Kidney	MK391608

## Discussion

The tropical climate, high average temperatures (30°C–36°C) and humidity of Malaysia favor the survival and proliferation of leptospires. In Malaysia, cats are the most favored and common companion animal living in close proximity to humans owing to cultural and religious beliefs. Urbanization of the countryside has led to an overpopulation of wild animals in the cities, including rats (*Rattus* spp.) and consequently, an increased likelihood of transmission of zoonoses, such as leptospirosis, to other stray animals as well as to humans [29]. In a previous study on shelter cats in Malaysia, the results showed a high sero-detection of anti-*Leptospira* spp. antibodies in the shelter cat population as high as 18% [30]. This indicates that cats in Malaysia are frequently, and hence pervasively, exposed to leptospires. Owing to the successive reports of high sero-detection of leptospiral infection in cats locally and globally, there is concern over the possibility of cats acting as asymptomatic reservoirs of this pathogen. Recent studies have shown that cats can shed the DNA from pathogenic leptospires in urine [13,14]. The aim of the present study was to elucidate several aspects of leptospiral infection in cats. More specifically, the objective first was to investigate the possibility of a cat acting as a reservoir for this pathogen. This investigation was done by using serological and molecular techniques to determine the shedding and carrier status of leptospires. Secondly, the study sought to investigate the viability of leptospires in cat urine by using culture and isolation methods. The role of cats as a potential source of leptospiral infection in humans and animals was investigated and the first isolation of Lepto*spira* spp. from urine and kidney samples of naturally and apparently healthy infected cats were reported.

Furthermore, the total detection for pathogenic *Leptospira* spp. infection in cats was 29.2% (n = 24/82) through the use of MAT, PCR, and/or culture techniques. Serologically, 25.6% (n = 21/82) of the cats had positive antibody titers (100–1,600) against the pathogenic serogroups, namely Bataviae, Javanica, and Ballum. Cats in this study had higher leptospiral sero-detection compared to that of other recent cats studies in the USA, Brazil, Iran, Canada, Taiwan, and Germany, which ranged between 4.8% and 17.9% [11–14,31–34], but lower sero-detection compared to those reported in Greece and Serbia, which ranged between 26% and 33% [35,36]. MAT test results may vary because of the serovar panel used in the geographical area of interest and the cut-off value. The high seropositivity detected in stray cats from these two shelters in this study is indicative of the high exposure of cats to leptospiral infection in Malaysia. Although serological detection methods have been extensively used for the diagnosis of anti-*Leptospira* spp. antibodies in cats and other species, its limitations must be taken into account during interpretation of the results. For example, using the test in the early stage of the infection can result in the absence or weak presence of anti-*Leptospira* antibodies in addition to a possible cross reaction to more than one serogroup [37]. PCR is a highly sensitive and specific test for detecting the DNA of microorganisms, including leptospires [38,39]. In the present study, by using PCR, leptospiral DNA was detected in the blood of seven cats. All the amplicons sequences were revealed as *L. biflexa*, which is a saprophytic species known to live in water and soil without infecting animals [40]. However, *L. biflexa* serovar Patoc was previously found in cats by using culture method [22]. In this study, serological examination revealed that there were no antibodies recorded against serogroup Patoc 1, whereas 28.5% (n = 2/7) of those cats were seropositive to serogroup Bataviae and one was culture positive for Bataviae. There may have been a concurrent exposure with Patoc, but no antibodies for it have been produced yet.

In the present study, PCR was a useful tool for detecting the leptospiral shedding in cats. The DNA from pathogenic leptospires in urine was detected in four cats (4.9%, n = 4/82). The findings revealed lower frequency rates compared to those of a study in Taiwan, which reported 80 out of 118 cats (67.8%) that had urinary DNA shedding of leptospires [14] and higher than in other studies in Germany (3.3%, n = 7/215), Canada (3.4%, n = 8/238), and more recently in Thailand (0.8%, n = 2/260) [12,13,41]. This could be attributed to the intermittent shedding of leptospires in the urine, as well as possible false negatives due to PCR inhibitors present in the clinical samples [42]. The possible false negative derived by using PCR in this study cannot be ruled out where all positive samples that showed leptospiral growth using culture test yielded negative results through PCR. Interestingly, all four cats that were found to shed leptospires DNA in their urine were also seropositive at titers of ≤200, thus indicating that cats with leptospiral infection are able to shed the pathogenic leptospiral DNA even at low serological titers. In a previous study, it has been suggested that even a low serological titer could be a reflection of an active leptospiral infection in cats [32]. In this study, the positive serological results obtained at titers of 100 were compatible with direct PCR on the urine samples and culture test (urine, kidney), which suggests that even low titers of 100 could be indicative of an active infection in cats. The findings of the study support the probability that cats can shed the pathogenic DNA of leptospires and may play a role in the transmission of the disease to humans.

Bacterial cultures revealed that 16.7% (n = 4/24) of the cats were positive carriers while both urine and kidney samples show that one cat was culture positive. MAT of hyperimmune sera and partial DNA identifications were helpful for identifying the leptospiral isolates from the cats. All the isolates were identified serologically as Bataviae and revealed as *L. interrogans* through the use of phylogenetic analysis based on 16s rRNA. Additionally, *L. interrogans* Bataviae is reported to be the most prominent and common isolated serovar in human, dog and rat studies in Malaysia [43–48]. Moreover, it is globally associated with the infection of multiple hosts and causes clinical signs in humans. The findings of the study revealed that a cat can act as a source of highly pathogenic leptospiral serovars to other animals and humans. Given the natural rat-hunting behavior in cats and an aversion towards water, the possibility of cats becoming infected through rat hunting cannot be ruled out. This infection host-scenario could be supported by knowing that Bataviae was the most isolated serovar from rats near locations to the cat shelters in this study [48]. This may raise concerns for the possibility of cats playing a role in the transmission from rats (the main source of leptospires) to humans. The isolation of leptospires can be challenging from both body fluids and tissue owing to the slow growth of leptospires and frequent contamination of clinical samples. Furthermore, the organism is excreted intermittently in the urine [49]. The culture test of leptospiral is a less sensitive method; this result thus suggests that carrier status among the cats could be more than 16.7%. Through the use of MAT in this study, three out of four culture-positive cats showed no serum antibody reaction towards Bataviae serogroup. It should be noted that the sera of these three cats were re-tested with the isolates as well and revealed no agglutination. This shows that cats could be infected even without showing any antibody production, as previously reported [14,50]. However, this is the first definitive confirmation of a pathogenic strain isolated from three cats without antibody production. Pathogenic leptospires were retrieved from cat urine and kidney samples in this study by using culture methods, and the finding supports that cats can shed the pathogenic leptospires and may play a role in the transmission of the disease to humans.

Renal involvement has been reported in seropositive cats for leptospires, and the most common clinical signs reported were polyuria and polydipsia [10,12]. In the present study, cats appeared emaciated with evidence of feline upper respiratory disease during the physical examination. Monitoring for polyuria and polydipsia was not possible as the cats were confined to a large cage in the shelters. Nevertheless, the correlation between kidney disorders and leptospiral infection in cats is still a subject of discussion [19]. The absence of signs that represent kidney and/or other signs of leptospiral infection in the cats in this study might indicate sub-clinical infection. The present study is the first report of pathogenic leptospires retrieved from urine and kidney samples obtained from naturally infected cats. The actual occurrence might be higher in reality, as false negative leptospiral culture results are not uncommon because of the difficulties in successfully obtaining isolates from cat urine. The MAT detection of leptospiral exposure and characterization of the isolated serovar helped to elucidate the epidemiology of the Bataviae serovar in cats.

## Conclusion

This study is the first report of pathogenic leptospires isolation from naturally infected cat urine and kidney samples locally and globally. The results of the study revealed high-frequency detection of leptospiral infection in cats in Malaysia. However, planned and region-based studies are warranted to provide valuable insights into the epidemiology of leptospirosis in the cat population of Malaysia. Our results provide evidence that healthy cats may serve as reservoirs of leptospires sub-clinically and sub-serologically, and this awareness may subsequently encourage the development of disease prevention strategies for the cat population. Future studies involving molecular investigation on the isolates from this study are warranted.

## Supporting information

S1 Data *Leptospira* spp. serovars used for microscopic agglutination test (MAT) in this study. (XLSX)

S2 Data Polymerase chain reaction (PCR) primers used in the PCR study. (XLSX)

S3 Data The hyperimmune sera panel used in microscopic agglutination test (MAT) characterization. (XLSX)

S4 Data Summary of the results of the cats against leptospiral infection in used tests in this study. (XLSX)

S5 Data Serological results of the cats against leptospiral infection in shelter 1 using microscopic agglutination test on set of 21 antigens’ panel. (XLSX)

S6 Data Serological results of the cats against leptospiral infection in shelter 2 using microscopic agglutination test on set of 21 antigens’ panel. (XLSX)

S7 Data Results of the cats against leptospiral infection in shelter 1&2 using polymerase reaction test with the sequencing results of 16s rRNA gene. (XLSX)

S8 Data Identification of retrieved isolates from cats’ urine and kidney samples, using the microscopic agglutination test (MAT) hyperimmune sera, and polymerase chain reaction (PCR) characterization. (XLSX)
